# Long term type 1 diabetes is associated with hand pain, disability and stiffness but not with structural hand osteoarthritis features – The Dialong hand study

**DOI:** 10.1371/journal.pone.0177118

**Published:** 2017-05-16

**Authors:** Karin Magnusson, Kristine Bech Holte, Niels Gunnar Juel, Jens Ivar Brox, Kåre Birger Hagen, Ida Kristin Haugen, Tore Julsrud Berg

**Affiliations:** 1 National Advisory Unit on Rehabilitation in Rheumatology, Department of Rheumatology, Diakonhjemmet Hospital, Oslo, Norway; 2 Clinical Epidemiology Unit, Orthopaedics, Department of Clinical Sciences Lund, Lund University, Lund, Sweden; 3 Department of Endocrinology, Oslo University Hospital, Oslo, Norway; 4 Department of Physical Medicine and Rehabilitation, Oslo University Hospital, Oslo, Norway; 5 Department of Rheumatology, Diakonhjemmet Hospital, Oslo, Norway; 6 The Norwegian Diabetics’ Centre, Oslo, Norway; 7 Institute of Clinical Medicine, Faculty of Medicine, University of Oslo, Oslo, Norway; University of Colorado Denver School of Medicine, UNITED STATES

## Abstract

**Objective:**

To explore whether having long-term type 1 diabetes (>45 years) is associated with a higher prevalence of radiographic hand OA, erosive hand OA and increased hand pain, disability and stiffness.

**Methods:**

In total N = 96 persons with type 1 diabetes diagnosed before 1970 were included (mean [SD] age: 62.2 [7.4], mean [SD] HbA_1c_: 7.43 [0.80] and N = 49 [51%] men). Regular measurements of their HbA_1c_ were obtained till 2015. We included N = 69 healthy controls without any diabetes (mean [SD] age: 63.0 [7.0], mean [SD] HbA_1c_: 5.41 [0.32], N = 29 [42%] men). The groups were compared for radiographic hand OA (Kellgren-Lawrence grade ≥2 in ≥1 joint) and erosive hand OA (central erosions in ≥1 joint), Australian/Canadian index (AUSCAN) for hand pain, disability and stiffness using regression analyses adjusted for age, sex, educational level and waist circumference.

**Results:**

We found no associations between having long term type 1 diabetes and more prevalent radiographic hand OA (OR = 0.83, 95% CI = 0.38–1.81). We found a trend towards higher prevalence of erosive hand OA in diabetes patients (OR = 2.96, 95% CI = 0.82–10.64). Strong and consistent associations were observed between long term type 1 diabetes and increased hand pain (B = 2.78, 95% CI = 1.65–3.91), disability (B = 5.30, 95% CI = 3.48–7.12) and stiffness (B = 2.00, 95% CI = 1.33–2.67). These associations were particularly strong for women and participants below the median age of 61 years.

**Conclusion:**

Long-term type 1 diabetes was not associated with radiographic hand OA, but was strongly associated with hand pain, disability and stiffness. The association between diabetes and erosive hand OA warrants further investigation.

## Introduction

Persons with hand osteoarthritis (OA) may experience pain and disability in daily life activities to a similar extent as persons with inflammatory joint diseases [1]. A good understanding of risk factors for OA is important for prevention since no disease-modifying drugs exist. Risk factors for OA may act through mechanical or systemic pathways on the joint [1,2]. Most previous studies of the etiology of OA have regarded the knee joint [3]. The knee joint is affected by high mechanical loading which may interact with obesity and related metabolic factors, making the study of isolated effects of each risk factor on the development of OA challenging.

The effects of mechanical risk factors are strongly reduced in the hand joints implying a less confounded study of systemic risk factors for OA [4]. Known risk factors for hand OA include female sex, higher age, genetic factors, previous joint injury and hypermobility, whereas the risk related to obesity and components of the metabolic syndrome such as dyslipidemia, hypertension and diabetes mellitus is still unclear [5,6]. A distinct diabetes mellitus OA phenotype has been proposed [7]. However, few studies have explored the association between diabetes mellitus and hand OA. A recent systematic review and meta-analyses showed a higher prevalence of diabetes mellitus (mainly type 2 diabetes) in persons with hand OA than in persons without [8]. The review included only two full-length articles of studies of the hand [9,10], showing an association between diabetes and hand OA particularly at younger ages [10]. Contrarily, a recent study of incident hand OA and type 2 diabetes showed no association [11].

There are several potential causal pathways through which diabetes may lead to hand OA and/or pain in hand OA. Diabetes may cause a low-grade systemic inflammation, and thereby contribute to hand-joint inflammation and pain [7]. Patients with long term diabetes may also suffer from diabetic neuropathy which may contribute to pain [12]. Furthermore, the increased formation of advanced glycation end products (AGEs) may contribute to OA by various mechanisms affecting collagen tissue and chondrocytes. The explanatory mechanisms linking diabetes mellitus to hand OA may further be dependent on the OA phenotype. We have previously shown that diabetes mellitus may be a potential cause of pain in erosive hand OA but not in non-erosive hand OA, supporting previous studies indicating a distinct metabolic/diabetes/erosive OA phenotype [6,7,13,14].

Previous studies have examined OA in persons with accumulation of several metabolic risk factors including type 2 diabetes, making it challenging to isolate the independent effect of long-term hyperglycaemia on the development of hand OA. The study of OA in a type 1 diabetes mellitus population allows for the study of the more pure effects of chronic hyperglycaemia on hand OA. Improved knowledge of the association between type 1 diabetes and hand OA/hand pain will provide important knowledge for development of preventive and disease-modifying interventions. Our aim was to explore whether having long-term type 1 diabetes is associated with a higher prevalence of radiographic hand OA, erosive hand OA and increased hand pain, disability and stiffness.

## Patients and methods

### Participants and data collection

The present study is a sub-study of the Dialong study, a cross-sectional, controlled, retrospective study of persons attending the Norwegian Diabetes Centre (NDC) from various time periods to 2015. The main aim of the study was to explore macrovascular and non-vascular complications in patients having long-standing type 1 diabetes for >45 years. Patients attending the NDC in 2014 with type 1 diabetes diagnosed before 1970 were invited to take part in the study as cases exposed to a long term high glycaemic burden (N = 136). The diagnosis was based on (i) clinical criteria (ii) lack of insulin production as measured by fasting c-peptide, i.e. <0.2 pmol/ml and (iii) HbA1c > 6.5%. The exposed cases were asked to invite their spouses or close friends without diabetes to participate as healthy controls in the study. Potential controls with known diabetes or undiagnosed diabetes as measured by HbA_1c_ > 6.5% were excluded as well as first degree relatives. We also excluded participants with known inflammatory rheumatic disease in the current study.

Data were collected at two different sites in 2015, the Oslo University Hospital, Ullevål (OUHU) and the NDC. During two visits, participants had fasting blood tests, underwent conventional radiographs of the hands, responded to questionnaires and had their height, weight and waist circumference measured. Participants also underwent a clinical examination of their hands with assessment of hand complaints as well as an assessment of the presence of peripheral neuropathy at one of the visits. All patients signed informed consent and the Regional Ethical Committee South East (Regional Committees for medical and health research ethics) approved the study.

### General diabetic assessment and measurement of glycaemic burden

We searched medical records at NDC and performed patient interviews to establish the date of diagnosis of type 1 diabetes and any co-morbidities. Most of the patients had attended the NDC since the time of diagnosis with extensive documentation of their diabetic control and complications. Longitudinal HbA_1_ and HbA_1c_ values were available from the early 1980s up untill 2015. In total, the mean (SD) number of measurements taken per subject at different time intervals was 73 (29).

Different laboratory methods were applied in different time series. One method was used for HbA_1_ (Agarose gel electrophoresis at Oslo University Hospital, Aker, Oslo, Norway) from1981 to 1986 and two different methods for HbA_1c_ (Diamat (Bio-Rad Laboratories, Hercules, CA) from 1987 to 1993 and DCA2000 analyzer (Bayer Diagnostics, Tarrytown, NY) from 1993 onwards). To transform old HbA_1_ for the earliest time series into HbA_1c_, we identified duplicate HbA_1_/HbA_1c_ values (taken on the same date) from 50 different subjects and found a regression formula to convert HbA_1_ to HbA_1c_ (HbA_1c_ = 0,526 + 0.776HbA_1_). We found excellent Intraclass Correlation Coefficient (ICC) of 0.94 (95% CI = 0.89–0.97) between the HbA_1_ and HbA_1c_ values, which is in accordance with previous findings [15].

We calculated time-weighted mean HbA_1c_ as a measure of glycaemic burden for the diabetes patients, similar to the large Diabetes Control and Complications Trial and Follow-up study (DCCT/EDIC) [16]. As we do not have HbA_1c_ values from time of diagnosis up until the 1980s, we created two different variables for each participant with long term diabetes: one that only incorporated existing values from the 1980s up till today and one that weighted the initial values with number of years from diagnosis till the first HbA_1c_ value (estimated full duration HbA_1c_). The last method better reflected the actual glycaemic burden as we incorporated all years living with the disease and we therefore used this mean time-weighted HbA_1c_ as a continuous measure of glycaemic burden in the analyses. Details of the calculations are presented in S1 Fig. All participants had HbA_1c_ taken at OUHU (High Performance Liquid Chromatography, reference range 4.0–6.0) in 2015 (current HbA_1c_). Microvascular complications as a result of long term type 1 diabetes, i.e. persistent albuminuria were defined as an albumin creatinine ratio of > 2.9 mg/mmol on two consecutive samples, including the sample taken in 2015.

### Conventional radiographs

The participants underwent conventional radiography (frontal images) of both hands. The bilateral first carpometacarpal (CMC-1), scaphotrapezotrapezoidal (STT), 1^st^-5^th^ metacarpophalangeal joints (MCP), thumb interphalangeal (IP-1), 2^nd^-5^th^ proximal interphalangeal joints (PIP) and 2^nd^-5^th^ distal interphalangeal joints (DIP) were scored for radiographic OA according to a modified Kellgren-Lawrence (KL) scale [17,18] and for central erosions according to the OARSI atlas [19] by one experience reader (IKH). The modification of the KL scale refers to the scoring of definite joint space narrowing as present OA also in the absence of osteophytes ([17,18]. Hand OA was defined as involvement of ≥2 hand joints with Kellgren-Lawrence grade (KLG) ≥2), whereas central erosion in one or more of the joints was required for erosive OA. After several weeks/months, 20 x-rays were re-evaluated. The ICC values for KL sum score (0.98, 95% CI 0.95–0.99) and number of erosive joints for the bilateral hands were excellent (1.00, 0.99–1.00).

### Hand pain, physical function and stiffness

Self-reported hand pain, disability and stiffness were assessed using the Australian/Canadian (AUSCAN) scale [20,21]. The scale consists of 3 subscales measuring 1) hand pain during rest and activity (5 items), 2) physical hand function during activities in daily life (9 items) and 3) hand stiffness (1 item). Each item is measured on a 0–4 numerical rating scale. Sum scores were calculated for each item and included as a continuous outcome variable in the analyses (0–20 scale, 0–36 scale and 0–4 scale, respectively). Higher score represents more pain, worse physical function and worse stiffness.

### Covariates

Educational level was reported in four categories ranging from primary school to college/university and dichotomized into low (upper secondary school) vs. high education level (college/university [0–1]). Height and weight were measured wearing light indoor clothing and Body Mass Index was calculated (kg/m^2^). We also measured waist circumference in centimeters. All participants had their systolic and diastolic blood pressure measured and the total cholesterol and triglycerides were obtained from fasting blood samples. The presence of hand diagnoses other than OA (i.e. carpal tunnel syndrome, trigger finger, Dupuytren’s contracture and limited joint mobility of the hands) was assessed according to a standardized procedure by an experienced specialist in physical medicine and rehabilitation (NGJ). We created a categorical variable indicating 0 to 4 hand complaints (0 was the ref. category). The presence of peripheral neuropathy (yes/no) was defined based on either previously confirmed diagnosis by a positive nerve conduction test or the presence of symptoms such as numbness, unsteadiness, aching, burning pain or pins and needles, as well as symmetrical signs in both lower extremities using standard monofilament and vibration tests.

### Statistical analyses

We performed secondary analyses of the Dialong study, which was initially powered to study the association between coronary artery disease and glucosepane, an advanced glycation end product in skin collagen. Hence, we had no possibility to increase the power a priori. For explorative reasons and due to previous findings with regard to erosive OA [13], we included erosive OA as an outcome despite the relatively low study sample. Prior to all analyses, we checked distributions of covariates and explored statistically significant group differences between the exposed and unexposed participants using t-tests and chi-square. To study the associations between diabetes and hand OA outcomes, we performed separate regression analyses using the presence of long-term type 1 diabetes (yes/no), the current HbA_1c_ for cases and controls and the estimated full duration HbA_1c_ (cases only) as continuous independent variables. The presence of radiographic OA and erosive OA (logistic regression), hand pain and disability (linear regression) as well as stiffness (ordinal regression) were included as dependent variables in separate models. We performed crude analyses, analyses adjusted for age and sex as well as fully adjusted analyses (adjusted for age, sex, educational level and waist circumference). Since persons with type 1 diabetes and proteinuria may be at higher risk of complications such as OA, we adjusted for persistent albuminuria (yes/no) in the analyses of full duration HbA_1c_ for cases only. We also additionally adjusted the analyses of the diabetes variables and AUSCAN hand pain, physical function and stiffness for hand complaints (carpal tunnel syndrome, trigger finger, Dupuytren’s contracture and limited joint mobility of the hands on a 0 to 4 scale) in separate analyses. Finally, we adjusted the analyses of AUSCAN hand pain for the presence of peripheral neuropathy to explore the likelihood of residual confounding. This was avoided in the main analyses since hand OA pain may be of both neuropathic and nociceptive origin.

We tested for statistical interactions by including a product term between the exposure (i.e. case-control status, current HbA_1c_ or estimated full duration HbA_1c_) and age and sex in the fully adjusted model (i.e. the model adjusted for age, sex, education status and waist circumference). If any statistically significant interactions (p<0.10) were found, we performed analyses stratified by age (above and below median) and sex (men and women). The stratified analyses were adjusted for sex, age, education level and waist circumference. We repeated the analyses using a continuous score representing the severity of radiographic hand OA (KL sum score, 0–120). Since metabolic factors may be associated with hand OA and HbA_1c_/glycaemic burden, we also repeated the analyses with additional adjustment for hypertension (systolic blood pressure ≥135 mm HG and/or diastolic blood pressure ≥85 mm HG), total cholesterol and triglycerides (continuous variables) in a sensitivity analysis. This additional adjustment was not done in the main analyses as it would imply a reduced statistical power (particularly for estimated full duration HbA_1c_ as the exposure variable and erosive hand OA as outcome variable) and a conditioning on variables that are functions of the selection process, which might open up spurious, biasing and non-causal paths. Prior to the regression analyses, we inspected plotted residuals to evaluate any deviation from a normal distribution (no strong deviations observed). Furthermore, all analyses were run with a robust standard error to account for potential heteroscedasticity. Results are presented as unstandardized beta estimates or Odds Ratios (OR) with 95% confidence intervals (CI). All analyses were performed using STATA MP v. 14. The minimal underlying dataset can be found in S1 Dataset.

## Results

Out of the N = 136 cases exposed with long-term type 1 diabetes, N = 105 agreed to participate. We excluded N = 9 due to psoriatic arthritis (N = 1), rheumatoid arthritis (N = 2), lack of hand joint examination (N = 2), lack of blood samples (N = 2) or withdrawal of consent (N = 2), leaving N = 96 persons for the analyses. In total N = 80 persons without diabetes were invited, representing healthy controls. Of these, N = 75 agreed to participate and they had no diabetes as tested by HbA_1c_. We excluded N = 6 persons from the diabetes-free control group due to spondyloarthritis (N = 1), psoriatic arthritis (N = 2), lack of blood samples (N = 2) or lack of hand joint examination (N = 1), leaving N = 69 for the analyses. Participants’ characteristics are presented in Table 1. The range of all participants’ HbA_1c_ measurements was 4.4–9.9 mmol/l whereas cases’ estimated full duration HbA_1c_ range was 5.8–9.8 mmol/l. Cases exposed to long term diabetes and the healthy controls had a similar age and sex distribution and had a similar education level, BMI and waist circumference, since there were no statistically significant differences between the exposed vs. the unexposed (p>0.05). The measured diastolic and systolic blood pressure, total cholesterol and triglycerides were significantly higher in healthy controls than in cases exposed to long term diabetes (all p<0.05).

**Table 1:** Participants' characteristics.

	Cases N = 96	Controls N = 69
Sociodemographic variables		
Age, mean (SD)	62.2 (7.4)	63.0 (7.0)
Sex, M, n (%)	49 (51.0)	29 (42.0)
Education level (high), n (%)	60 (62.5)	51 (76.1)
Anthropometrics and metabolic covariates		
Waist circumference, mean (SD)	91.7 (13.0)	89.4 (12.9)
Body Mass Index, kg/m^2^, mean (SD)	26.2 (4.0)	25.8 (4.3)
Systolic blood pressure (mm HG), mean (SD)	146.4 (19.9)	136.9 (19.9)
Diastolic blood pressure (mm HG), mean (SD)	75.2 (8.3)	80.9 (9.5)
Hypertension [0–1], n (%)	43 (62.3)	79 (82.3)
Total cholesterol (mmol/L), mean (SD)	5.0 (1.0)	5.8 (1.2)
Triglycerids (mmol/L), mean (SD)	0.9 (0.4)	1.1 (0.6)
Diabetes variables		
HbA_1c_ in 2015, mean (SD)	7.43 (0.80)	5.41 (0.32)
Glycaemic burden (i.e. mean time-weighted HbA_1c_), mean (SD)	7.95 (0.81)	
Years lived with type 1 diabetes, mean (SD)	50.7 (4.8)	
Persistent albuminuria, n (%)	17 (18.1)	
Peripheral neuropathy, n (%)	62 (64.6)	14 (20.3)
Osteoarthritis variables		
Kellgren-Lawrence sumscore [0–120], median (IQR)	10 (3–21)	12 (6–21)
Radiographic OA [0–1]), n (%)	60 (62.5)	44 (63.8)
Non-erosive OA [0–1], n %)	49 (81.2)	41 (93.2)
Erosive OA [0–1], n (%)	11 (18.3)	3 (6.8)
AUSCAN pain [0–20], mean (SD)	4.1 (4.9)	1.4 (2.6)
AUSCAN physical function [0–36], mean (SD)	7.6 (8.0)	2.6 (4.4)
AUSCAN stiffness [0–4], mean (SD)	1.3 (1.0)	0.4 (0.6)

Abbreviations: M: Males, SD: standard deviation, AUSCAN: Australian-Canadian Hand Index, OA: osteoarthritis. Radiographic OA: ≥2 joints with Kellgren-Lawrence grade 2 or more. Erosive OA: ≥1 joint with central erosions.

### Radigraphic hand OA

We found no association between being a case exposed to long-term type 1 diabetes or having a higher current HbA_1c_ and having one or more joints with radiographic hand OA (Table 2). Among the cases exposed to long-term type 1 diabetes, there was also no association between higher glycaemic burden and more frequent radiographic hand OA (Table 2). Similarly, no associations were found between the diabetes measures and increasing severity of radiographic hand OA using the KL sum score (range 0–120) as outcome variable (B_exposed case_ = -0.69, 95% CI = -5.00–3.62, B_HbA1c_ = -1.30, 95% CI = -3.02–0.43, B_glycaemic burden_ = 0.05, 95% CI = -4.30–4.40). Additional adjustment for persistent albuminuria in the analyses of exposed cases only did not change these results (B_glycaemic burden_ = -0.07, 95% CI = -4.45–4.31). Furthermore, the exposed cases with higher glycaemic burden did not have higher risk of radiographic hand OA or erosive hand OA when additionally adjusted for persistent albuminuria (OR = 0.89, 95% CI = 0.44–1.73 and OR = 0.89, 95% CI = 0.44–1.73, respectively).

**Table 2:** Associations between case-control status, HbA_1c_, glycaemic burden and structural hand OA features.

	Crude OR (95% CI)	Age and sex adjusted OR (95% CI)	Fully adjusted OR (95% CI)
Outcome: Radiographic OA			
Exposed case (ref.: non-exposed)	0.95 (0.50–1.80)	1.08 (0.54–2.12)	1.11 (0.55–2.26)
HbA_1c_ in 2015	0.87 (0.66–1.15)	0.91 (0.69–1.22)	0.92 (0.68–1.25)
Glycaemic burden	0.84 (0.49–1.45)	0.92 (0.53–1.59)	0.91 (0.47–1.77)
Outcome: Erosive OA			
Exposed case (ref.: non-exposed)	2.85 (0.76–10.66)	3.07 (0.83–11.29)	2.96 (0.82–10.64)
HbA_1c_ in 2015	1.12 (0.80–1.56)	1.16 (0.80–1.66)	1.15 (0.79–1.67)
Glycaemic burden	0.88 (0.38–2.02)	0.88 (0.37–2.13)	0.91 (0.31–2.61)

Logistic regression analyses (fully adjusted analyses were adjusted for age, sex, education status and waist circumference). KLG; Kellgren-Lawrence Grade, Glycaemic burden; mean time-weighted HbA_1c_ (available in exposed cases only), OA; osteoarthritis, OR; odds ratio, CI; confidence intervals. Radiographic OA: present KLG≥2 in ≥2 joints. Erosive OA: present erosions in ≥1 joint.

There was a trend towards a higher prevalence of erosive hand OA in persons with long-term type 1 diabetes, although this tendency was not statistically significant (Table 2). No statistically significant interactions were found between any of the diabetes measures and age or sex for neither radiographic nor erosive hand OA. Additional adjustment for hypertension, total cholesterol and triglycerides did not change any results (data not shown).

### Hand pain, physical function and stiffness

Contrarily to what was observed for radiographic and erosive hand OA, we found strong and consistent associations between having long-term type 1 diabetes, higher current HbA_1c_ levels and estimated full duration HbA_1c_ and increased hand pain, reduced physical function and increased hand stiffness (Table 3).

**Table 3:** Associations between case control status, HbA_1c_ and hand pain, function and stiffness.

	Crude B (95% CI)	Age and sex adjusted B (95% CI)	Fully adjusted B (95% CI)	Fully adjusted, +adjusted for hand complaints B (95% CI)
Outcome: Hand pain (0–20 scale)^a^				
Exposed case (ref.: non-exposed)	2.65* (1.49–3.81)	2.88* (1.76–3.99)	2.78* (1.65–3.91)	1.59* (0.34–2.83)
HbA_1c_ in 2015	1.42* (0.87–1.95)	1.51* (0.99–2.02)	1.43* (0.91–1.95)	1.06* (0.50–1.62)
Glycaemic burden	2.16* (1.05–3.28)	2.19* (1.19–3.19)	2.00* (0.77–3.19)	1.76* (0.49–3.03)
Outcome: Hand disability (0–36 scale)^a^				
Exposed case (ref.: non-exposed)	5.04* (3.11–6.97)	5.65* (3.88–7.43)	5.30* (3.48–7.12)	3.77* (1.75–5.80)
HbA_1c_ in 2015	2.30* (1.45–3.16)	2.55* (1.78–3.32)	2.40* (1.60–3.20)	1.84* (0.92–2.76)
Glycaemic burden	3.00* (1.20–4.80)	3.15* (1.57–4.72)	3.15* (1.37–4.94)	2.84* (0.99–4.70)
Outcome: Hand stiffness (0–4 scale)^b^				
Exposed case (ref.: non-exposed)	1.90* (1.27–2.52)	2.07* (1.42–2.73)	2.00* (1.33–2.67)	1.39* (0.65–2.12)
HbA_1c_ in 2015	0.93* (0.65–1.21)	0.98* (0.71–1.26)	0.92* (0.63–1.21)	0.72* (0.40–1.03)
Glycaemic burden	0.65* (0.27–1.03)	0.76* (0.32–1.21)	0.67* (0.10–1.24)	0.50 (-0.14–1.15)

^a^ Linear regression analyses,

^b^ ordinal regression analyses. Cases exposed to long-term type 1 diabetes (N = 96), non-exposed are healthy controls (N = 69). Fully adjusted analyses were adjusted for age, sex, education status and waist circumference. Fully adjusted +adjusted for hand complaints were additionally adjusted for the presence of the hand complaints carpal tunnel syndrome, trigger finger, Dupuytren's contracture and limited joint mobility on a 0–4 scale. Glycaemic burden; mean time-weighted HbA_1c_ (available for exposed cases only), B; unstandardized Beta estimate, CI; confidence intervals.

* implies p<0.05.

The estimates were reduced after adjustment for other hand diagnoses, but the diabetes measures remained statistically significantly associated with hand pain, stiffness and function (Table 3). However, the association between glycaemic burden and AUSCAN stiffness in cases only was not any longer statistically significant after adjustment for hand complaints (Table 3). When the analyses of AUSCAN hand pain including adjustment for other hand diagnoses were additionally adjusted for the presence of peripheral neuropathy, the estimates remained more or less similar (B_exposed case_ = 1.39, 95% CI = 0.02–2.77, B_HbA1c_ = 1.01, 95% CI = 0.41–1.63, B_glycaemic burden_ = 1.74, 95% CI = 0.41–3.07).

Additional adjustment for persistent albuminuria in the analyses of estimated full duration HbA_1c_ in cases only did not change the results (in analyses also adjusted for age, sex, education and waist circumference; B = 1.96, 95% CI = 0.74–3.19, B = 3.13, 05% CI = 1.31–4.94, B = 0.67, 95% CI = 0.10–1.23 for AUSCAN pain, physical function and stiffness, respectively. To avoid over-adjustment, these analyses were not adjusted for other hand diagnosis nor peripheral neuropathy). For all symptom measures, there were statistically significant interactions between age and sex for current HbA_1c_ as well as for case/control status. In stratified analyses on age below and above median (61 years) we found a stronger association between having long-term type 1 diabetes, higher HbA_1c_ levels and increased hand pain, reduced physical function and stiffness in the youngest participants compared to the elderly (Table 4). We further found stronger associations in women than in men for all outcomes (Table 4). We observed a significant statistical interaction with age in analyses of glycaemic burden as the independent variable and hand pain as the dependent variable in analyses of the exposed cases only. Stratified analyses showed a stronger association in participants below median age whereas there was no association in participants above (B = 2.77, 95% CI = 1.06–4.49 and B = 0.51, 95% CI = -1.31–2.34, respectively). No age or sex differences were observed for physical function or stiffness when using glycaemic burden as the exposure variable (p>0.10 for product term). Additional adjustment for hypertension, total cholesterol and triglycerides did not change any of the observed associations (data not shown).

**Table 4:** Age and sex stratified analyses for the association between long term type 1 diabetes and hand pain, disability and hand stiffness.

	AUSCAN pain B (95% CI) ^a^	AUSCAN physical function B (95% CI) ^a^	AUSCAN stiffness B (95% CI) ^b^
Case exposed to diabetes vs. healthy control (ref.)			
Women	4.18 (2.35–6.00)*	7.66 (4.64–10.67)*	2.78 (1.68–3.89)*
Men	0.94 (-0.25–2.13)	2.05 (0.52–3.58)*	1.10 (0.18–2.03)*
<61 years	4.48 (2.05–6.91)*	6.90 (3.32–10.48)*	2.59 (1.32–3.86)*
≥61 years	1.57 (0.26–2.88)*	3.79 (1.64–5.95)*	1.59 (0.74–2.43)*
Exposure: Current HbA_1c_			
Women	1.88 (1.05–2.71)*	3.31 (1.93–4.68)*	1.20 (0.75–1.66)*
Men	0.91 (0.26–1.55)*	1.27 (0.49–2.04)*	0.63 (0.21–1.05)*
<61 years	2.03 (1.25–2.82)*	2.88 (1.64–4.12)*	1.17 (0.74–1.60)*
≥61 years	0.75 (0.06–1.45)*	1.63 (0.55–2.71)*	0.65 (0.24–1.06)*

^a^ Linear regression analyses,

^b^ ordinal regression analyses. Cases exposed to long-term type 1 diabetes (N = 96), non-exposed are healthy controls (N = 69). Estimates are from analyses adjusted for age, sex, educational status and waist circumference (Hence, sex-stratified analyses were adjusted for age, and age-stratified analyses were adjusted for sex as well as residual confounding by age). AUSCAN; Australian-Canadian Hand Index, B; unstandardized Beta estimate, CI; confidence intervals.

* implies p<0.05.

## Discussion

In the current study we found no association between having type 1 diabetes and radiographic hand OA. However, we found a tendency towards a higher prevalence of erosive hand OA in patients with type 1 diabetes as compared to healthy controls. Long-term type 1 diabetes and higher full duration HbA_1c_ i.e. increased glycaemic burden were strongly and consistently associated with increased hand pain, a reduced physical function and stiffness. These associations were particularly strong for women and participants below the median age of 61 years.

Strengths of the study were the extensive measurements of HbA_1c_ in persons with type 1 diabetes giving a detailed estimate of total glycaemic burden. The cases had been exposed to a high glycaemic burden over >45 years and the inclusion of healthy controls with a similar age and sex distribution allows for comparison to persons without the exposure. To our knowledge, the current study is the first study of type 1 diabetes and OA, allowing for a more isolated analysis of the independent effect of chronic hyperglycaemia as measured by HbA_1c_ levels that is not confounded through other metabolic factors.

We could find no previous study of type 1 diabetes and OA for comparison of our findings. However, if disease mechanisms due to increased HbA_1c_ levels are similar in type 1 and type 2 diabetes (i.e. not mediated through, or affected by other metabolic factors), our study both contradicts and supports previous findings. A recent systematic review and meta-analysis found a significantly higher frequency of diabetes in OA patients than in persons without OA [8]. Based on only 3 studies, significant associations to hand OA (OR = 1.31, 95% CI = 1.07–1.61) were observed [8]. The included studies for the meta-analyses considered mainly cross-sectional prevalence data and the (type 2) diabetes could also be hypothesized to be an effect of a sedentary lifestyle due to co-occurring knee and hand OA [22]. Unlike the review, a recent large population based study of incident hand OA in 27.000 subjects found no associations to hand OA defined by primary care records [11], which is in accordance with our findings. Although relying on fewer participants, we were able to distinguish between structural OA damage and patient-reported pain, disability and stiffness showing marked differences in observed associations. To our knowledge, the dependency on age and sex for hand pain, disability and stiffness has not been previously observed.

There was a tendency to a higher prevalence of erosive hand OA among the cases exposed to long term type 1 diabetes (Table 1), which is in accordance with our previous findings for self-reported diabetes [13]. In contrast, none of the 34 patients with erosive hand OA in the Oslo hand OA cohort had self-reported diabetes, whereas 6/31 (19%) of the patients with non-erosive disease reported to have diabetes [23]. Both studies are hampered by small study samples. Erosive hand OA in the general population is rather uncommon with a prevalence of 9.8% and 3.6% in women and men between 40–84 years, respectively [18]. With an erosive OA prevalence of n = 3/69 (4%) (in controls) and n = 11/96 (12%) (in cases) in the current sample, we might have included too few participants for performing any robust analyses of the association between type 1 diabetes and erosive hand OA and the potential associations between long term type 1 diabetes and erosive hand OA should be further studied in larger samples.

The increased prevalence of hand pain, disability and stiffness in the present group of patients with long term type 1 diabetes was not explained by radiographic hand OA. Similarly, although the magnitude of the estimates was greatly reduced, long term type 1 diabetes was significantly and consistently associated with hand pain, disability and stiffness even after adjustment for the diagnoses: carpal tunnel syndrome, trigger finger, Dupuytren’s contracture and limited joint mobility of the hand. These complaints have previously been shown to have increased prevalence in persons with diabetes [24] and their prevalence in the Dialong study population will be described elsewhere (work in progress). The estimates for AUSCAN hand pain were also only slightly attenuated after adjustment for peripheral neuropathy, implying other, unmeasured factors might explain the association between type 1 diabetes and the AUSCAN variables. As an example, we had no information on e.g. present joint inflammation, peripheral or central sensitization. Furthermore, psychological factors might have influenced our findings. Hence, future studies should further explore explanatory factors for the strong associations between long term type 1 diabetes and increased hand pain, reduced physical hand function and increased stiffness.

Our study had some weaknesses. First, we had too low power to detect any statistically significant differences between cases and controls for erosive hand OA. Our post hoc power analyses revealed that N = 133 participants per group would be required to detect any difference with the observed proportions affected. For radiographic hand OA in ≥2 joints, we observed equal proportions affected in the exposed versus unexposed group. N = 79 subjects per group would be required to detect a 20% difference in prevalence with 80% power at a 5% significance level. Similarly, as an example for KL sum score, indicating amount and severity of radiographic OA ranging from 0 to 120, N = 86 participants per group would be required to detect a difference of 6 units (with observed SD 14 and 80% power at a 5% significance level) [25]. For AUSCAN pain and physical function, the differences between exposed and unexposed were greater than proposed cut-offs for minimally clinically relevant differences (1.49 for AUSCAN pain and 1.25 for AUSCAN physical function) [26], and we concluded we had sufficient power for all outcomes except erosive hand OA. A second weakness of the study is that the cases attending the NDC having HbA_1c_ measures available for calculation of glycaemic burden may have a slightly less severe disease than persons not attending. The cases were compared to the National Norwegian Diabetes Register showing persons with type 1 diabetes in the same diabetes duration and age as our participants had a mean HbA_1c_ of 7.6% in 2015 [27]. With a mean current HbA_1c_ of 7.4% in our study we might have underestimated the true associations. The selected controls, who were spouses and friends to the patients, may also not be representative to a general population, introducing additional selection bias. We observed a significantly higher blood pressure, total cholesterol and triglycerides among controls than among cases, which might be due to the more frequent use of HMG-CoA reductase inhibitors by the exposed cases (53.3% vs. 16.2%, respectively). However, according to standard epidemiological methods, a control group should not be free of exposure, but similar to the population the researcher wants to make inference about. As an example, the blood pressure and BMI of the controls was similar to, or slightly lower than that observed in a study population representative for the Norwegian population [28], which we believe minimizes the risk of skewed selection having biased our results. A third weakness of our study is our calculation of time-weighted mean HbA_1c_. The methods for measuring HbA_1_/HbA_1c_ were developed around 1980. Our first values are therefore taken in the early stages of the study with probable lower validity and reliability before an international standard of measurement was established (the US based “National Glycohemoglobin Standardization Program” (NGSP) in 1996). To compensate for this, we calculated the average of the years without HbA_1c_ readings from the first three years rather than the first single reading. We therefore cannot say whether our calculations are over- or under-representing the actual HbA_1c_ values up until the 1980s. Nevertheless, we believe giving more weight to the early values taken before the universal introduction of intensive insulin-treatment, better reflects the full duration of diabetes. A final limitation of our study was the lack of data on occupation. Some studies indicate people with work tasks involving extensive use of the hands might be more affected by hand OA [29]. However, the high level of education in both study groups makes this assumption less likely.

In conclusion, the current study showed that long term type 1 diabetes was not associated with radiographic hand OA, but was strongly associated with increased hand pain, hand stiffness and reduced hand function, particularly in women and young participants with higher HbA_1c_. The association between diabetes and erosive hand OA should be further explored using larger sample sizes.

## Supporting information

S1 Fig Equations for calculating Mean Time-Weighted HbA_1c_ for each individual (MTW) and Full Duration Mean Time-Weighted HbA_1c_ for each individual (FDMTW). (DOCX)

S1 Dataset Minimal data set underlying the findings in the study. (DTA)
