# Supplementary material for: Long term type 1 diabetes is associated with hand pain, disability and stiffness but not with structural hand osteoarthritis features – The Dialong hand study
Source: PLoS One. 2017 May 16;12(5):e0177118. doi: 10.1371/journal.pone.0177118 (PMC5433713; doi:10.1371/journal.pone.0177118)
Supplement: S1 Fig — (DOCX) [file pone.0177118.s001.docx]

S1 Fig

**Equations for calculating Mean Time-Weighted HbA_1c_ for each individual (MTW) and Full Duration Mean Time-Weighted HbA_1c_ for each individual (FDMTW).**

For “Long Term Type 1 Diabetes as Associated With Hand Pain, Disability And Stiffness But Not With Structural Hand Osteoarthritis Features – The Dialong Hand Study”

By Magnusson et al., 2017.

$$\boldsymbol{MTW}=\frac{1}{n}\sum_{i=1}^{n} (\frac{1}{m}\sum_{i=1}^{m} X_{HbA1c i})$$

$$\boldsymbol{FDMTW}= \frac{\left( \left( \frac{1}{3}\sum_{i=1}^{3} \left( \frac{1}{m}\sum_{i=1}^{m} X_{HbA1c i} \right) \right)\times(Year of 1st {HbA}_{1c}-Year of diagnosis) \right)+(MTW\times n)}{Duration of type 1 diabetes in years}$$

Where *n* is the number of years between the first and last HbA_1c_ tests, the last test having been carried out in 2014, *m* is the total number of HbA_1c_ tests per year and *X_HbA1c i_* is the observed value for test *i*

To calculate MTW, we first calculated the mean HbA_1c_ for each year, and then the mean of these values.

To calculate FDMTW we calculated the average HbA_1c_ from the first three years it was measured in the individual, and multiplied this by the number of years which have elapsed between diagnosis and the first measurement of HbA_1c_ (Estimated HbA_1c_). We then multiplied the MTW by the number of years of HbA_1c_ readings, added that figure to the Estimated HbA_1c_ and then divided the total figure by the number of years with diabetes.
